# The Lived Experience of Caregiving and Perception of Service Provision among Family-Caregivers of People with Late-Stage Parkinson's: A Qualitative Study

**DOI:** 10.1155/2023/4483517

**Published:** 2023-02-03

**Authors:** Joy Read, Sarah Cable, Gergely Bartl, Charlotte Löfqvist, Susanne Iwarsson, Anette Schrag

**Affiliations:** ^1^Department of Clinical and Movement Neurosciences, UCL Institute of Neurology University College, London, UK; ^2^Department of Health Sciences, Lund University, Lund, Sweden

## Abstract

**Background:**

The complex nature of late-stage Parkinson's requires multiagency support and leads to an increased burden on family members who assume a multiplicity of responsibilities. The aim of this study is to further understand the lived experiences of family-caregivers and their perception of, and satisfaction with, service provision.

**Methods:**

This qualitative substudy was a part of the European multicentre Care of Late-Stage Parkinsonism (CLaSP) project. Purposive sampling resulted in a sample of eleven family-caregivers of people with late-stage Parkinson's, who were interviewed using semistructured open-ended questions. Thematic analysis followed. *Findings*. Three overarching themes were developed from the data: ensuring continuous support is vital to providing care at home, perceiving unmet service provision needs, and advocating and co-ordinating all aspects of care take their toll. These themes include not only experience of services that caregivers find supportive in order to deliver care but also of disjointed care between multiple agencies, a perceived lack of Parkinson's expertise, and there was a lack of anticipatory future planning. The constancy and scope of the family-caregiver role is described, including the need to project manage multiple aspects of care with multiple agencies, to be an advocate, and to assume new roles such as managing finances. Multiple losses were reported, which in part was mitigated by gaining expertise through information and support from professionals and organised and informal support.

**Conclusion:**

The intricacies and consequences of the family-caregivers' role and their experience of service provision indicate the need to acknowledge and consider their role and needs, fully involve them in consultations and provide information and joined-up support to improve their well-being, and ensure their continuous significant contribution to the ongoing care of the person with Parkinson's.

## 1. Background

The complexity and multifaceted nature of advancing Parkinson's requires ongoing primary care, specialist services, and increasing long-term support from multiagency health and social services. In addition, increased use of secondary care services with increased risk of hospitalization follows falls or infections [1], often precipitating care-home placement [2]. Whilst at home, there is an increased reliance on family members, frequently a spouse, adult offspring, or sibling [3], who become caregivers providing physical, social, domestic, and emotional care in the home, for up to 16 hours per day [4]. The caring demands related to reduced mobility and multiple fluctuating, incapacitating, and upsetting nonmotor symptoms (NMSs) of Parkinson's have been shown to negatively affect the physical, social, financial, psychological, and quality of life (QoL) of family members [5–9], with a higher burden compared to caregivers of older adults in general [10].

Parkinson's has no clear trajectory or duration, and its resulting dependency can therefore vary in years from diagnosis to death [11], with a range of between 1 and 21 years of caregiving reported [4, 10]. In addition to duration, caregiving strain increases and QoL reduces by increased age and health needs in caregiving-spouses [12], and working-age offspring may be navigating life stage demands on top of caring demands. It has been found that, across conditions, such informal (unpaid) care had an estimated value of £132 billion annually in 2015, almost double its value in 2001 [13]. Despite caregiver strain being well documented, especially in relation to the burden of specific Parkinson's symptoms including significant impairment and falls; behavioural and cognitive changes; and neuropsychiatric symptoms of depression, apathy, and impulse disorders [5–9], and the roles and key activities assumed [4, 10], there is limited knowledge about the lived experience of family-caregivers in relation to their perception of service needs and provision for those with late-stage Parkinson's who have high degrees of disability. It is important to understand in depth the caregivers' experience and perception of service provision to comprehend the gaps they perceive and therefore often fill. Addressing unmet needs can potentially equip and support carers in multifaceted ways to continue offering support [14–16]. This is of broad significance as evidence suggests that caregiver strain is a strong predictor for the selection of institutionalised care for those with later-stage Parkinson's [17, 18], and the largest direct cost of Parkinson's is typically nursing home costs and inpatient care [19].

These are important considerations given the extensive provision of care by family-caregivers. Also given the predicted increase in prevelance and global burden due to Parkinson's [20]; and that the population is aging and Parkinson's occurs in people over the age of 60 [21]. Gaining insights into the experiences of family-caregivers and their perceptions of service use is therefore warrented. The findings are of value to help support those caring for people with Parkinson's and also applicable to family-caregivers of other progressive neurodegenerative conditioons. The aim of this study was therefore to facilitate an in-depth exploration and further comprehend the lived experience of caregiving for late-stage Parkinson's and the perception of service needs and provision from the family-caregivers' perspective in England.

## 2. Methods

### 2.1. Design

This study had an explorative qualitative approach, using semistructured interviews [22] with family-caregivers. Interview data were analysed using thematic analysis [23], and reported guidelines by the Standards for Reporting Qualitative Research framework (COREQ) [24].

### 2.2. Ethics

The study was granted ethical approval from Camden and Kings Cross Research Ethics Committee, London (IRAS 143636.14/LO/0367). Written informed consent was obtained from all participants.

### 2.3. Sampling and Participants

Participants were purposively sampled [25] to obtain a breadth of ages, genders, living situations, and disability of the person with Parkinson's (PwP); they were providing care for from the English cohort (*n* = 123) of the European “Care of Late-Stage Parkinsonism” (CLaSP) study [26].

Participants in the present study (*n* = 11) were family-caregivers of those with late-stage Parkinson's (findings reported elsewhere [27]). The PwP were caring for had been diagnosed according to UK Parkinson's Disease Society Brain Bank clinical diagnostic criteria [28], for at least seven years, and with disease severity stage 4 or 5 during the “On” state on the modified Hoehn and Yahr Scale (H&Y) [29, 30], or significant disability indicated by a score of 50% or below on the Schwab and England scale [31]. As analysis took place alongside the interview process, recruitment ceased once there was confidence that saturation was reached [32], that is, saturation was identified as attained when additional interviews did not reveal any new, extra information related to the study aim.

The recruitment process was closed when the sample consisted of 11 family-caregivers, the majority of whom were female spouses, living in their own homes with the PwP in urban or suburban areas in and within a 50-mile radius of London, England. In one case, their partner with Parkinson's was residing in a nursing home. The sample included three daughters and one sibling (Table 1).

### 2.4. Procedure

Participants were recruited through general practitioners' (GPs) surgeries, NHS hospital outpatient clinics, Parkinson's charities, and specialist neurologists in and within Greater London, England. Initial recruitment approaches were made by clinicians, where invitation letters and information sheets were given to potential participants who were asked to return reply-slips to the research team should they wish to take part in the study. Following confirmation of eligibility, and the chance to ask questions, written informed consent was obtained.

Interviews used a study topic guide specifically for family-caregivers (Table 2) which was based on study objectives and developed by members of the CLaSP consortium involved in the qualitative arm of the project (see also [27]) and was further refined during application. Open-ended questions explored the perceived impact on life situations, needs, opinions about care and services, personal challenges and the positives of being a family-caregiver, deficits and barriers to care provision, and future care decisions. Prompts and probes were used, and responses were summarised to ensure that the information revealed during the interviews was correctly understood.

The interviewers had healthcare and/or psychology backgrounds. They were further trained for the study by qualitative methods experts, and therefore had the skills to build rapport and encourage information sharing during the interviews. All but one interview took place at the participant's place of residence, with one interview taking place in a private room in a nursing home. In all cases, interviews took place between the interviewer and the participant alone so that open discussion was facilitated. This included the eight instances where both the family-caregiver and PwP participated in the CLaSP qualitative study (PwP findings using a PwP-specific topic guide reported elsewhere: [27]). Interviews took an average of 60 to 90 minutes, were recorded using a digital recorder, transcribed verbatim by the interviewers, deidentified and data stored securely. The interviews presented below use a unique study ID. Interviews took place in a process separate from the quantitative data collection for the main CLaSP study [26], and the qualitative interviews and analysis took place over a period of twelve months during 2016.

### 2.5. Analysis

Content thematic analysis [23] was applied to identify, analyse, and describe themes as suggested by Clarke and Braun [23], and data were managed using NVivo 11 Pro [33]. An inductive approach was taken, and transcripts were read repeatedly by the first and second authors (JR and SC) to build an overview of all content and develop initial coding lists, concentrating on the study aims. The separate code lists were compared (JR and SC) and combined to create a coding frame, which was reviewed (CL, SI, and AS) and applied line-by-line to all data of interest (JR and SC). Codes were discussed throughout the process with all authors, and previously coded data were revisited (JR and SC) whenever new codes were identified. Themes and the definition of categories and subcategories were developed, and interpretations were discussed regularly with all authors throughout the analysis process to ensure validity. Trustworthiness was ensured by constantly reviewing the raw data, and supportive and reflective quotes were identified and selected.

## 3. Findings

In late-stage Parkinson's, the family-caregivers' lives become increasingly focused on the needs of the PwP and interactions with service providers, which changed the shape and content of the family-caregivers' lives, as reflected in three overarching themes, each with two to three subthemes shown in Table 3, and supported by additional quotes in the Supplementary Table (available here) entitled “Exemplar quotes.”

### 3.1. Ensuring Continuous Support Is Vital to Continue Providing Care at Home

When family-caregivers spoke about “having needs met” this was rarely about their own specific needs, but instead related to those of the PwP which if met had benefits or positive consequences for the family-caregivers. The supports that enabled family-caregivers to continue providing home-based care without excessive impact on themselves were through *making use of multiple sources of support to manage life with Parkinson's* and *continuously finding the right information is vital to becoming an expert.*

#### 3.1.1. Making Use of Multiple Sources of Support to Manage Life with Parkinson's

Family-caregivers felt responsible for managing often deteriorating situations, and support with this was seen as coming through the availability, efficiency, and good relationships with known local service providers, specifically Parkinson's Disease Nurse Specialist (PDNS), social workers, and occupational therapists (OTs). Their professional input of specialist knowledge including about medications and symptom control, about financial support, and the provision of specialist equipment such as hoists or bathing aids, helped ensure the needs of the PwP were appropriately met. This subsequently supported family-caregivers, as the provision of equipment such as a wheelchair meant the couple could go out of the home and maintain some social contact. Similarly, the interview data revealed that changes in the PwP behaviour benefitted from specialist input for appropriate management which facilitated the family-caregiver continuing to providing care at home, and appropriate advice helped relieve the sense of responsibility and isolation:

“*She is on the other end of the phone and I can you know say*, (*name of specialist nurse*)” *I am desperate*.” And she will say “*Right I will be over tomorrow morning*” if I can sort of survive (...) if I have any problems over medication, I just give her a ring and ask her to come over or we go and see her in the clinic or something such as this and talk, talk the medication through (1094).

In addition, to support from health professionals, organisations in the voluntary sector facilitated couples remaining in their homes through the provision of assistive equipment, and importantly their service identified appropriate workers to assist with home maintenance; a task that for many elderly couples would otherwise be difficult or prohibitively expensive. Moreover, practical and emotional support for family-caregivers also came from other family members, longstanding friends, and the wider community, including faith communities and neighbours:

“We have lovely neighbours as well who have been over in the night to help me once when (PwP) fell. It was one in the morning, I went and knocked on their door and, immediately (neighbour) came over, you know. Picked (PwP) up. Took him up to bed and tucked him up” (1094).

Support was also found through Parkinson's and generic caregivers support groups, which provided emotional support and a “safe space” to share the experience of being a family-caregiver. In a couple of cases family-caregivers took on organisational roles within both Parkinson's and community support groups, providing them with purpose, responsibility akin to previous employment, and respite away from personal caregiving demands: *“and I go to that on my own and that helps me”* (1064). A break from caregiving responsibilities was also found when the PwP spent time elsewhere for respite care, which was sometimes organised with the help of social workers or on the advice of PDNS, although often accompanied by a sense of guilt in the family-caregiver, was restorative and sometimes seen as essential for family-caregivers.

#### 3.1.2. Continuously Finding the Right Information Is Vital to Becoming an Expert

Family-caregivers felt they carried the ultimate responsibility of providing or managing care and the interview data revealed that they either recognised the importance of becoming experts on the needs of the PwP and about Parkinson's, or became experts out of necessity, and that ongoing information was specifically identified as important in equipping them. Ongoing information was required as the condition advanced, particularly as information provided early on had often been forgotten or needs unexpectedly changed. Relevant information was obtained through healthcare professionals, through research and charity websites for example Parkinson's UK, and some support groups offered structured information and facilitated experiential information from peers, which was reported as being of value:

“Well, it is all Parkinson's patients with a Parkinson's nurse and they usually have a subject and it could be diet, constipation, and exercise. And they have an hour or so with coffee and biscuits and things so you can talk” (1094).

### 3.2. Perceiving Unmet Service Provision Needs

Family-caregivers spoke about the current service provision they received as being *experiencing fragmented and insufficient care for a complex condition* and that there was a *lack of anticipatory planning for the future*.

#### 3.2.1. Experiencing Fragmented and Insufficient Care for a Complex Condition

The family-caregivers described the care system as being complex, fragmented, inefficient, inflexible, overstretched, and understaffed, resulting in negative outcomes for those with Parkinson's and stressful ramifications for themselves. Family-caregivers described that care delivery was predominantly community-based, varied geographically, and service structure meant that there was a range of professionals and agencies that they had to liaise with. These included care agencies and formal carers, a variety of nurses including district, practice, elderly care, and PDNS, also social workers, OT's, physiotherapists, falls teams, and secondary care including neurology outpatients, and hospices. The result of which often resulted in fragmented care and additional burden to family-caregivers:

“He was getting all this sort of fragmented bits of care that were not the kind of coming together (…) a meeting to bring it all together. (…) I think I was trying to get some more care. Or was I trying to organise some respite? I was very tired” (1103).

Lack of specialist care was highlighted as problematic, with some not wishing to contact certain professionals feeling that they would not understand or be able to provide the required symptom management. In addition, it was felt that some formal carers did not have an adequate understanding of Parkinson's, thus creating discomfort about the care delivered:

“Some of the girls (formal carers) are lovely and some of them are absolutely diabolical. The problem is with the carers is that they do not really understand about the Parkinson's. They clearly had no training on it. You know some of them have come in and they even, they do not mean to do it nastily, but where my dad goes “b, b, b, ba, and b” (carer demonstrating the difficulty the PwP experienced when speaking) they take the mickey out of him and go “b, b, b come on (name), b, b, and b” “(carer demonstrating how formal carers mimicked the speaking pattern of the PwP), and repeat it back. They do not even realise how cruel that is” (1071).

#### 3.2.2. Lack of Anticipatory Planning for the Future

Managing daily life often consumed family-caregiver time and energy meaning the focus was primarily on the present rather than the future, and the unpredictable symptoms and uncertain disease trajectory also meant that managing and adapting to the immediate were more practicable. Some who would prefer to look ahead to the future were, however, inhibited by the PwP: *“Mum does not like to think about the future”* (1106), whilst others were in environments, usually a hospice, where future considerations were encouraged:

“And they do things like living wills and end of life care (…). So, that is very good (…) it makes you think (…) rather than just thinking about them in your head, actually verbalising them a bit which is a very good idea actually! So, and I think some people, well you all shy away from it a bit do not you because it is the last thing you really want to be thinking about” (1094).

The preference and intention of many family-caregivers were to continue looking after the PwP at home, nevertheless, future institutional care was often viewed as inevitable, despite previous poor experiences: “*The poor Parkinson's sufferers were trying to eat, cut their food up, and could not, and they were just whipping the plates away*” (1064); or concerns about the financial complexities and a lack of clarity or support in how to manage that: “*So social services were saying that it was a nursing need and nursing were saying that it is a social need”* (1071); or promises made to the PwP: “*You will never put me into a care home will you?” and so, of course, I say “no I will not,” I mean I can't say “yes one day I will,” because that would be horrible really*” (1064); or the fear that the person would deteriorate once admitted: *“I think he will go in there and he will nose dive”*(1071).

Despite any current difficulties, participants still deferred the decision for care to take place other than at home as a future option rather than something to consider in the present. Instead, decisions became superfluous when admissions to a nursing home became unavoidable, for example, in response to unmanageable deterioration, or when increasing demands were in conflict with commitments such as needing to work in paid employment or care for other family members: “*And I have spoken to me dad about it and I have said to him, “I have got to put my kids first, same as you would have put me first, you have got to go*”(1071). In these two examples, social services became involved in finding suitable alternative accommodation, however, participants did not speak about other health professionals or services advising or becoming involved in any preparation for the future.

### 3.3. Advocating and Co-Ordinating All Aspects of Care Take Its Toll

The consequences of fragmented care from multiple care agencies meant that family-caregivers had to be an advocate for the PwP and co-ordinate all aspects of care, described under the theme of *assuming a project manager role.* A consequence of this was that they were never off duty, described under *managing a constancy of demands*, which led to *perceiving personal loss.*

#### 3.3.1. Assuming a Project Manager Role

In the absence of co-ordinated services, many family-caregivers assumed the roles of advocate and “project manager,” planning, organising, and directing external agencies to deliver the best possible care for the PwP. This demanded family-caregiver time, energy, and knowledge in liaising with multiple agencies, including communicating with those responsible for the formal care provision, for example, care agency managers, so that care was delivered, and service deficits were effectively addressed. Given that many participants were from the postwar generation, deference to medical professionals was evident, however, the driving needs of the PwP meant many became proficient in communicating with medical professionals and health agencies to ensure needs were met, and if met also improved the life of the family-caregiver.

Perhaps because they were “on-hand” and enmeshed in the PwP everyday needs or they felt that tasks were inappropriate for other family members, spouses often filled gaps in daily care provision, for example, attending to hygiene needs if a formal carer failed to keep an appointment. In contrast siblings and adult offspring often relied on care agencies to facilitate their external employment or to fulfil other responsibilities. Siblings and offspring, rather than spouses, described the need to “monitor” the care provided, sometimes describing external care as substandard with care staff arriving late, or missing visits, not understanding Parkinson's, or not respecting the PwP: “*they (carers) constantly talk over him*” (1071), or delivered inadequate care:

“I expect the same standard as I give him. And it is not always the case and so, because I am here I can see what they are giving him as a care company and what I give him and it is not the same and it is frustrating. So, I am constantly chasing them and that is tiring” (1095).

For several, decreased income and increasing demands on the household budget needed to be managed, including purchasing assistive equipment or supplies unavailable through other routes. Financial complexity associated with progressing symptoms meant family-caregivers assumed the role of “financial director” navigating external financial systems often without much support or guidance, and for some managing finances for the first time due to the PwP reduced ability to continue managing these. Such financial systems included applying and managing grants, for example, to create a walk-in shower-room, navigate the benefit system to claim disability living allowance, and manage payment of formal carers:

“They put in the money every month and we put in money every month into that account and all the carers get paid from that account. So, it is a separate account for the care” (1106).

These “project manager” activities were in addition to being sole initiators and facilitators for any social contact, or responsibility for responding to medical emergencies requiring external help, and to managing all areas of household life, which previously might have been managed as a couple or by the PwP him-/herself, putting increased demand on family-caregivers:

“And there is still the shopping and the washing, everything that they do not, that they do not class as being, because he is with family it is our duty. Our duty as a family is to look after him. (…) It is a nightmare it really is, it takes up all my time dealing with stuff” (1095).

#### 3.3.2. Managing a Constancy of Demands

Providing care and support for the well-being of someone with late-stage Parkinson's at home meant that family-caregivers increasingly felt they were “never off duty,” especially those living with the PwP as *“It is twenty-four hours because you are always on the lookout for one thing or another*” (1059). The constancy of demands was present throughout the day and night, with symptoms such as sleep disturbance and nocturia negatively impacting the family-caregivers sleep and therefore daytime function. Some caring activities were planned, for example, helping the PwP to dress or eat, but at all other times family-caregivers had to be mindful of spontaneous needs, for example, helping with toileting, or preventing or responding to falls; and the unpredictability of symptoms also made planning difficult. In addition, the progression of the condition meant there was a significant change in pace, where certain tasks became increasingly time consuming, for example, swallowing difficulties in the PwP meant feeding or administering medications consumed a substantial amount of family-caregiver time. Consequently, family-caregivers became less free to fully pursue their own activities or household tasks.

The interview data revealed that other members of the family described feeling similar pressures resulting in some being torn between the PwP and their children's needs, for example, with a burdensome constant responsibility, described by a sibling:

“I have got to be the driver and I cannot mentally and physically take that on. Umm otherwise my life gets consumed. I feel that it gets consumed now” (1095).

#### 3.3.3. Perceiving Personal Loss

The constancy of demands, deficits in service provision, and increased care requirements meant family-caregivers experienced losses in multiple areas of their lives. For some, there was a loss of “space,” independence, friendships, ability to travel, spontaneity, interests, “the life they had,” and a “loss of self”:

“A sense of loss of my own life, I would love to regain a sense of myself, it is the mantra which I repeat to myself, (…) you are also entitled to a life, although your life will have been changed irreparably because you have a partner you care for, (…) but you too are entitled to something that is yours too. Achieving that is very difficult” (1021).

For spouses, there was a diminishing of the relationship with the PwP. This was due to the reduced physical ability to share activities and socialize together but also changes in behaviour and loss of cognitive ability in the PwP altered the relationship dynamic. For example, impaired memory meant that decisions previously made as a couple had to be taken by the family-caregiver alone. There was an ambivalence about how much it was appropriate to discuss with wider family members and friends, which increased feelings of isolation. The sense of isolation was further increased when friendships and contacts with other family members were reduced or lost, often due to the burden of additional tasks limiting time and capacity, the PwP becoming anxious when the family-caregiver left home, or difficulties arranging and paying for alternative care for the PwP to facilitate time away from home.

Despite reduced contact, solace and support were often provided by wider family members with examples of them providing advice, assisting with household maintenance, information provision, and grandchildren being the following: “*part of keeping me sane*” (1059). Nevertheless, not all were nearby and were considered to have their own lives to lead:

“Unfortunately, our daughters, are all away, the help that they can give me is not as much as if they were able to do if they were living closer. But, you know they have their own lives. They do what they can. (…) I wouldn't expect them to be spending all their time here, that would not be right” (1064).

The interview data revealed that a loss of self was compounded by invariably adapting their own needs around those of others. Inadequate service provision meant a loss of career for some and the associated connections, satisfaction, identity, and financial renumeration. The latter having a negative impact on household finances. In addition, some family-caregivers had their own health issues, often ignored and sometimes exacerbated by the stress of their situation.

In order to mitigate the losses experienced family-caregivers felt it was important to maintain external interests and contacts where possible, facilitated by long-term friends and family members who understood if last minute changes to plans were necessary:

“I was going to meet one of my friends and I had to say, “you know, I cannot do that tomorrow (PwP) is not so brilliant at the moment” (1064).

## 4. Discussion

Through the three themes, developed from the interview data for the present study, of the following: (1) ensuring continuous support is vital to providing care at home, (2) perceiving unmet service provision needs, and (3) advocating and co-ordinating all aspects of care take their toll, the study extends the existing literature by presenting the lived experience and perception of service needs and provision from the perspectives of family-caregivers of those with late-stage Parkinson's. It elucidates how the lives of caregivers significantly and detrimentally changed as their multifaceted caregiving role expanded with the progression of Parkinson's symptoms in the person they cared for and in relation to service provision. Whilst there is a growing understanding of the impact of caregiving on various aspects of caregivers' lives across the stages of Parkinson's [5–7, 34, 35] little qualitative work has been conducted on the experience of caregiving specifically in relation to service provision in late-stage Parkinson's.

As findings show there are multiple sources of professional and informal support available to caregivers, and that good relationships with service providers were important in ensuring both delivery of good care together with support and guidance which could be seen to enhance a sense of control and empowerment. A necessary consequence of being a family-caregiver was “becoming an expert” about the personal needs of the PwP, and the multiple aspects of Parkinson's management and care options, necessitating the learning of new skills often with minimal guidance, and highlighting the importance of ongoing information. This subsequently provided a sense of control in an otherwise out-of-control situation, and the importance of empowering careers through increasing their knowledge and focusing on their assets has been reflected in the findings of interviews with health care professionals [36].

Family-caregivers perceived that care provision was fragmentated, inefficient, and inflexible, and that services were overstretched. This subsequently led to negative outcomes for those with Parkinson's and stressful ramifications for family-caregivers as they took on additional tasks and responsibilities. In addition, there was a lack of required community-based specialist knowledge, as also reported in multiple sclerosis (MS) [37], and whilst PDNS, OTs, and social workers were cited as having significant supportive roles their availability varied geographically. The importance of regular access to specialist health care echoes findings from the Swedish CLaSP qualitative substudy cohort [14]. The need for more ready access to appropriate expertise and the need for specialist community psychiatric support is similarly indicated as in line with earlier literature reviews participants described behavioural and neuropsychiatric problems as being particularly difficult to manage [38, 39], and quantitative findings from all site data of the European CLaSP cohort showed that neuropsychiatric features were most strongly associated with caregiver burden [40]. A lack of community-based support and difficulties in PwPs attending clinical appointments, geographical differences in local healthcare provision, and a paucity of community-based specialist neuropsychiatric support may mean that telemedicine, previously explored in Parkinson's [41] and accelerated by the Covid-19 pandemic [42] could improve access to specialist services for family-caregivers of PwP in the late stages.

Elucidated by our findings, the demands of daily life and an uncertain disease trajectory and ambiguous future meant family-caregivers primarily focused on the present rather than future long-term care or end of life. This was unless hospice facilities were used, which perhaps reflects the difficulties in gauging when and who bears responsibility for introducing such conversations [43], suggesting a need for improved inclusion for palliative care and future planning conversations in consultations involving family-caregivers. Nevertheless, when the future was discussed, home-based care was the preferred long-term option, as reported elsewhere [44], although institutionalised care was seen as inevitable; a well-reported outcome for other chronic conditions but particularly Parkinson's [45] and echoed by PwP themselves in the accompanying article from the CLaSP study [27].

Mirroring the carers UK report [13], our findings confirm the high levels of informal care family-caregivers provide; however, in contrast to other studies describing the extent of practical nursing and medical activities undertaken everyday by caregivers [10], our findings provide insights into the extent the family-caregivers role interfaces with service providers when the condition is more advanced. This is often due to fragmented care necessitating the family-caregivers to either provide care or oversee, co-ordinate, and manage the multiple care providers to ensure the best care provision. This is of interest given that many cares will be older and may have their own deteriorating health to consider.

Previous questionnaire data have shown that caregiver strain exists across all stages of Parkinson's however accumulates as the disease progresses [46]. In this current data, the increasing disabling symptoms in PwP meant family-caregivers not only managed constant care demands and management of services but also had to be continuously vigilant to ensure safety, as reflected in other quantitative findings [4] and data from all sites of the European CLaSP cohort where caregivers reported spending 7.6 (+8.2) hours per day supervising the PwP [40].

Findings describe the multiplicity of the family-caregivers remit as many took on new roles such as managing family budgets including complex financial matters such as payment of formal carers and navigating the benefits system to claim disability living allowance. Some financial and household tasks might previously have been managed as a couple or by the PwP, providing an insight into the evolutionary and dynamic nature of caregiving responsibilities and reshaping of the family-caregivers' lives, as reflected in the dementia literature [47]. The tasks taken on by caregivers were acknowledged and appreciated by those with Parkinson's in this substudy reported elsewhere [27] and they described the reshaping of roles as both positive and negative.

The needs of the family-caregivers became subsumed with the needs of the PwP, so that support to deliver good care or good care provision, perceived as receiving a tailored service and responsive and approachable professionals, resulted in indirect benefits for themselves. For example, facilitating a wheelchair could result in positive consequences by being able to be social as a couple away from home. The importance of such “normalcy” in those with Parkinson's and other long-term conditions has been reported [48, 49]; however, family-caregivers are often cited as the facilitators of this “normalcy” in Parkinson's [50] and dementia [51] rather than being able to pursue that goal for themselves and consequently experience many losses, including social and employment, in their own lives.

In contrast to other studies [52, 53], participants did not discuss esteem-based benefits of caregiving such as giving their lives meaning or pride in their successes as caregivers. Instead, they spoke about losses in multiple internal and external life domains. Caregiving demands had significant social and personal impact consequently eroding personal time and challenge the management of their personal lives and routines, as reported in other advanced chronic illnesses [54]. Loss of personal relationships included with the PwP and friends, but relationships' losses were also societal when unable to interact with the world through careers, travel, and interests. As reported in MS [55] family-caregivers described neglecting their own interests and needs, thus compounding a sense of isolation and loss. Given the extent of personal loss and reliance on the family-caregivers' capacity to cope, a means of evaluating family-caregivers QoL, support networks, and mental well-being is advisory.

### 4.1. Implications

The study findings have several implications for clinical practice. Findings show the need for the multiple service agencies to provide more joined-up care and to help project-manage care, and support family-caregivers in meeting the constant demands and overcome the personal losses they experience. The importance of including family-caregivers in all consultations as increasing symptoms dictate home-based care, relying on family-caregiver resilience particularly as preparation for the future and end of life becomes more significant. During such consultations, family-caregiver well-being should also be evaluated and appropriate support considered. Given the significant role of family-caregivers in the provision of care, future care delivery will need to accommodate a potential gap due to the rise in one-person-households. Future longitudinal research could, for example, focus in more detail on the experience and perception of preparation and support for end of life care.

### 4.2. Strengths and Weaknesses

The sample was from a group who were providing care for those with significant disability meaning they are not easily recruited to research, thus providing important insights. The sample size was determined based on the saturation principle [32], implying that the inclusion of participants ceased when no new information was gained from additional interviews. Recruitment approaches meant that urban and suburban settings were included, with a variation of socioeconomic contexts within a 50-mile radius of London, England, providing an informative picture of residence and service availability. Despite this, there was a lack of ethnic diversity limiting the transferability of the findings to such population segments. Similarly, there are limitations to transferability of findings as although a purposive sampling approach was taken, the majority of characteristics were based on the person with Parkinson's rather than the family-caregiver. Despite Parkinson's being over-represented in men, the inclusion of predominantly female caregivers is problematic, especially as females tend to assume important supportive roles in marital relationships and in illness. However, the cohort offers some breadth as it includes spouses, adult offspring, and siblings.

Notwithstanding, the team of authors represents clinical and scientific expertise including neurology, nursing, psychology, occupational therapy, and gerontology. Following the rigorous methodology, researchers collaborated in an iterative process during the process, which served well to ensure the validity and trustworthiness of findings.

## 5. Conclusion

In the absence of appropriate and comprehensive accessible service provision, this study illustrates how family-caregivers are key providers of personalised care, and co-ordinators of multiagency care. As illustrated by the findings, this primary role is an ongoing personal challenge for family-caregivers, even whilst managing their own health problems or competing demands. Health and social care service providers need to be aware and responsive to the demands on family-caregivers and offer appropriate collaborative support for their crucial input to continue.

## Figures and Tables

**Table 1:** Participant characteristics (family-caregivers), *n* = 11, and characteristics of the persons with Parkinson's they cared for.

Demographic details of participants (family-caregivers)	
Gender	
Women (*n*)	10
Men (*n*)	1

Relationship with person with Parkinson's	
Spouse (*n*)	7
Daughter (*n*)	3
Sister (*n*)	1

Living arrangements	
Spouse living with a person with Parkinson's (*n*)	6
Spouse living alone. Person with Parkinson's residing in a nursing home (*n*)	1
Family member living separately but visiting regularly (*n*)	3
Family member living with a person with Parkinson's (*n*)	1

Characteristics of person with Parkinson's	
Duration since PD diagnosis	
Range (years)	8–27
Mean (years)	17

H&Y stage	
Stage 4 (n)	4
Stage 5 (n)	7

Age	
Range (years)	70–88
Average (years)	78

Education	
Range (years)	8–16
Average (years)	12

**Table 2:** Family-caregiver interview topic guide.

Interview with family-caregiver living at home with a person with Parkinson's	Interview with family-caregiver of a person with Parkinson's living in a nursing home
(i) Personal needs and meeting of needs	(i) Needs and meeting of needs for both parties
(ii) Opinion about professional care received	(ii) Opinion about the residential care facility (including staff competence and medication)
(iii) Impact on personal health and life situation	(iii) Influence on care provided at the facility
(iv) Availability and opinion of respite services	(iv) Decision-making process to relocate to institutional setting (when, by whom, reasons, and information available)
(v) Opinion of residential care facility/nursing home, and if considered as a future option	(v) Feelings related to the move, at the time and in the present
(vi) Reasons current care situation	(vi) Management of care prior to the relocation and what would have been necessary to continue living there
(vii) Personal challenges in caring	(vii) What is missing?
(viii) Positive aspects of caring	(viii) Personal challenges
	(ix) Positive aspects

**Table 3:** Family-caregiver themes.

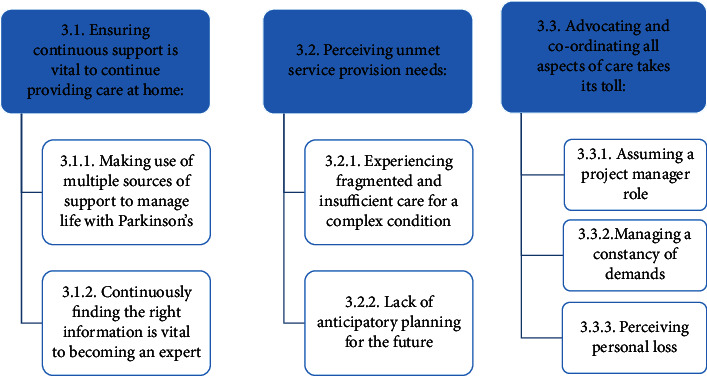

## Data Availability

The coding frameworks developed from the qualitative data used to support the findings of this study are available from the corresponding author upon request. Due to the confidential nature of qualitative data, the source data (i.e., interview data or transcripts) are not available as in order to protect participant privacy consent has not been given for individual data to be shared outside the direct research group.

## Additional Points

What is known: The ageing population with the increased prevalence of Parkinson's and the complexity of advanced Parkinson's imply that there is an increasing demand for health care and social service provision. Family-caregivers have a central role in facilitating care at home. As a consequence of taking on a breadth of caring duties to manage and respond to advancing Parkinson's symptoms, there is a considerable burden on family-caregivers which can negatively impact their lives and well-being, and the marital relationship. What this study adds: Family-caregivers of those with Parkinson's assume not only caregiving roles but also the “project-management” of many aspects of health care and social service delivery. The study provides insights into the extent the family-caregiving role interfaces with service providers, including when family-caregivers perceive the need to assume an advocacy role and to manage and monitor external care providers. The lives of family-caregivers are impacted not only by the symptoms of the person they care for but also by the structure and provision of care.

## Supplementary Materials

Supplementary Materials Supplementary Table 1: This includes exemplar quotes.
