# Supplementary material for: The Lived Experience of Caregiving and Perception of Service Provision among Family-Caregivers of People with Late-Stage Parkinson's: A Qualitative Study
Source: Parkinsons Dis. 2023 Feb 3;2023:4483517. doi: 10.1155/2023/4483517 (PMC9918353; doi:10.1155/2023/4483517)
Supplement: Supplementary Materials — Supplementary Table 1: This includes exemplar quotes. [file 4483517.f1.docx]

**Supplementary table 1: Exemplar quotes**

| **Theme 1., Ensuring continuous support is vital to continue providing care at home:** | |
| --- | --- |
| **Subtheme** | **Exemplar quotes** |
| **Making use of multiple sources of support to manage life with Parkinson’s** | “*When we were going away a couple of years ago and I thought we were at the stage of needing a wheelchair, we went through the NHS, and we had a wheelchair within ten days”. [1021]*  *“Most of it, it’s, I say most of it, it’s me saying “[PD nurses name] I’m a bit worried about”, or saying to the physio “what shall I do?” and she’ll say “well I’ve got a leaflet but I would suggest that you try this, this, this and this. And I have some equipment which will help you”. So when we had a problem with turning over in bed for instance they suggested umm er a slide sheet and they got me two”.* (1094)  *“I know she’s quite enjoyed going because they do things like hand massage and she’s come back with her nails painted, and she’s enjoyed chatting to people, or there, you know, because she’s quite sociable. She likes going out talking to people and if it was easier to get her, if it were once a week and if she was able to get there then it would be really good for her mentally, it would be very good. So that’s one recommendation I would make, they should have regular Parkinson’s groups*”. (1106)  “*She hasn’t got extra rails in the bathroom ‘cos she. Oh she’s got a thing over the bath, umm a special chair thing that goes up and down into the bath. Umm, but she’s got extra supports in the bedroom on the bed. A thing that makes it rise. So um she does have a regular assessment both for the flat and of her physical health and needs. And so yes they do look into her social and wellbeing, and mental health as well. Yes, so they do a full assessment*”. (1106) |
| **Continuously finding the right information is vital to becoming an expert** | “*I think other people are also, as I say a great source of info. If you can be talking to people who are in a similar position to you”.* (1059)  “*Well I suppose things like, welll [PD nurse] gave me a lot of hints, well not hints, a sort of general information about watching things like constipation now which is a big thing. I mean it sounds so petty but it’s such an enormous thing isn’t it*? *Yes, I suppose we’ve had quite a lot of information. Mainly from the Parkinson’s nurse, and going to these odd meetings and things. Trying to think what else. [persons name] has read up on the computer, early on, always, because he does, you know looks everything up. Yep we have, we’ve had a lot of stuff given to us. Booklets and things like this*”. (1094)  “*I do a lot of online research and there was this thing called a toto, which is a thing that turns you slightly in bed. Because I can’t turn him in bed. […] So, so more googling. I researched an all-terrain wheelchair which we’ve now bought, so now we have an all-terrain one. Which is [patients] wheelchair in there”*. (1103)  *“You don’t know what’s coming around the corner, you just don’t know. You’re constantly learning, you’re constantly adapting, you’re constantly making adjustments and you hadn’t got a clue*! […] *I’m an addictive reader and every time something happens I look it up, so I’ve done a lot of reading about it, but much of it’s observational, so both. If you read about it then see it. I have a living model that I can study twenty four hours a day!”* (1021)  “*But her main tablet is Madopar, and yeah it helps her. It doesn’t solve anything, but without it I’m sure she would grind to a halt. That’s what I think the medics say don’t they, that if you don’t take anything your quality of life would be zero. I mean (persons name) thinks her quality of life is zero now but I say look you may not think it’s clear but the tablets are helping a bit*”. (1064)  *“Well you’re the person who’s got to deal with this, at the end of the day you’re the expert”.* (1103) |
| **Theme 2., Perceiving unmet service provision needs:** | |
| **Experiencing fragmented and insufficient care for a complex condition** | “*They haven’t got a clue its Parkinson’s. They can’t seem to comprehend that he couldn’t feed himself. Some days he can’t feed himself. you look at me dad and you think “umm he’s not too bad is he?” And that’s what I get off them, but they’re only here for ten minutes each day so they assume”.* (1071)  “*I didn’t really have a social worker, as such. Until I started to ring at Age Concern and say, you know, I’m getting desperate*”. (1088)  *“The care, and people turning up on time. A major thing with me, for [patients] care is the morning carer turning up at a certain time. The carers company don’t seem to understand, I know he’s not the only one, but they don’t seem to understand that, so that um we’ve got a nine thirty slot so that could be either half an hour each side of nine thirty or later depending on who’s not turned up. You know if they’re short staffed. So they just think it’s acceptable that someone comes, it doesn’t matter who it is at least someone is coming at eleven O’clock, well, eleven OClock that means that [patient] is not having his medication and his breakfast until twelve”.* (1095)  “*And one of the other issues is when I’ve spoken to them, the organiser whoever it was, I said “is there somebody who could come, is there any transport?” you know could a transport person come and get her from her flat and help put her into the wheelchair and take her over. There isn’t. It doesn’t exist*”. (1106)  “*The two social workers I’ve had have been fantastic, but again this is just the luck of the draw, I had one who came in two days ago and I said “Oh that bed’ll need moving” because I knew at that point he couldn’t be nursed in it, because you can’t get a hoist in there. So said “You’ll have to move that. So I said “you’ll have to move that bed from that room to that room”. “Oh we can’t, I don’t think we do that” I said, “yes you do” I said “I can’t dismantle a specially err adapted bed and resemble it”. “But I’ve never heard of that before” she said*”. (1071)  “*They came and assessed him and they rejected him, they wouldn’t take him. Well then I had a call back from here (nursing home) saying that they can’t take him because it’s all dementia. Then Yes, they said he needed too much nursing care, they hadn’t got the availability to look after him*”. *Not enough staff. And that was here as well, because they haven’t really got enough staff. This is the point everywhere*”. (1088) |
| **Lack of anticipatory planning for the future** | “*Ignorance is bliss, because who wants to read that’s how you’re going to end up*”? (1071)  “*I would like [persons name] to be able to live at home, have as much a normal life as he possibly can* […*] where that is no longer possible and of course we will have to cross that line when we get to that*. *I could see that in the future what would work out best for us would probably be having one person who is almost like a housekeeper / carer who comes in for several hours of the day, so that person helps out, we know, we feel comfortable with, she feels, or he feels, comfortable with us, and it’s a repetitive thing, and while they’re here if (persons name) is sleeping they can prepare vegetables, they, you know, they can do simple things. And you know that would probably be the ideal, and sometimes people can achieve that, I know people who do and sometimes people don’t! […] I think finding someone like that is not easy, it really is not! “* (1021)  *“Whatever point in your life you are at I don’t think you really would like to think that “I’m eventually going to end up in a care home”, do you? I think most people would like to think, this sounds a bit morbid, but you leave this life in your own bed, and you won’t wake up one morning. And well that sounds quite nice doesn’t it*?” (1064)  “*I was going out of my mind. I had another call the next day, from a social worker, and he said “I think I can get him into (care home name)”. So that was it. He won’t come home I don’t think. He was saying to me in the latter few months before all this happened, “I know I’ve got to go into a nursing home permanently. You aren’t going to keep coping”. He had accepted that”* (1088)  “*We were discussing things like end of life care and all that sort of thing. And um you know they were saying “well you might not be able to keep him at home” you know when it really gets to that stage. Obviously I thought “Oh yes I will” Yes I bloody well will!” sort of thing You know not quite like that but you know that’s my determination! The GP came round, lovely GP, Dr [name] and he said “You know you should really think about, this is too much for you, you should really think about a care home for [patient]. And also my friends had been saying “it’s too much for you, A care home, you, you can’t go on like this and you find your. I found myself saying “No I’m not going to do that” you know. And it’s just a decision that grows. You know, and he knew me, he knew where he was and I thought “No he’s lost everything. I’m not going to have him in a care home. He’s not going to go into a care home. […] Because at the start you don’t know there’s going to come a time when the person can’t climb the stairs, or the person can’t do this. You don’t think about it”. (1103)* |
| **Theme 3. Advocating and co-ordinating all aspects of care takes its toll** | |
| **Assuming a project manager role** | “*Again, I have contact for people. There is a [borough] adult therapy team and I can contact them directly […] We have NHS, we have [borough] health care, right so, normally [borough] healthcare organise their staffs jointly with social services through […], through the particular agencies”*. (1103)  *“I sometimes talk to people and they say things like “Oh we really had to struggle to get you know the OTs to come or to get the physios to come. Well we didn’t seem to have any of that. Whether it’s because, I know it’s partly to do with the fact that we know how to talk to doctors and what not and it does help doesn’t it when you can explain over the phone what’s going on and how and when, and you know how to access people”*. (1094)  *“Well I’ve actually got in touch with someone who’s above the rest of them and she’s been very helpful. Since I’ve been communicating with her and things have got done whereas before I was complaining and it was, nothing was happening. It was going, it was getting lost in the system. Guess they didn’t want their managers to know that someones complaining. So since I’ve been speaking to [manager] she’s got things moving and it’s more on an even keel now*”. (1095)  “*And I’m now involved in the kind of campaign to try and get them to stop diverting the attendance allowance to local authorities, because that’s going to be a disaster*” (1103)  “*And then it’s up to you ie., me which carers I get, where I go to get the carers and who the carers are. The social services department at [council] don’t actually provide carers. They give you a list of agencies. Yeah so it’s then up, it’s down to the family to arrange it*”.(1106)  *“We get glitches perhaps every six months, or nine months where I can’t cope with the behaviour, so then I go and get someone and they tweak his medication […] I would think that I’m not going to be able to carry on if he behaves like this.”* (1038) |
| **Managing a constancy of demands** | “*The biggest problem for me, […] I would say the lack of sleep. If you’ve not had enough sleep and there are some days when I’ve had an hour and a half, sometimes more, anything less than four hours sleep and those four hours are often broken, not even solid, then it is impossible to function the next day*” (1021)  *“Ok, you have some free time, because he’s sleeping most of the time! But for example, he fell asleep in his chair in his office which is very comfortable, and although he can be very quiet I did hear him because he slipped out of the chair onto the floor – he was just wedged there on the floor – and he hasn’t done this for a long time but he’s done it twice in the last week”* (1021)  *“It’s like trying to get [*PwP*] to take his tablets this morning. He’s supposed to take them about eight and this morning his mouth was just ridged. So, I kept trying and eventually he took them about ten past nine*. […] *The way to go is keep going back and asking. It’s like getting him to drink – he won’t drink or he won’t drink enough. And you can offer him a drink and he’ll just turn it down. So you have to keep trying. Some mornings [persons name] does not want to know and he goes rigid, which is a Parkinson’s thing. You know he just goes rigid and he will not let them touch, he will not let them do anything. Umm mornings like that I say just leave him, we’ll deal with it later. Give him an hour and he’s totally compliant, and it’s very much Parkinson’s”.* (1059)  *“I mean we are in separate rooms because there is no way I could cope with this 24 hours a day. 16 hours a day fine but you know*!” (1064)  “*He had a sort of fit one night and I had to get the ambulance at midnight.*” (1094) |
| **Perceiving personal loss** | “*I don’t think we had the life we had. I think we are lucky in some ways, because of friendships and family, but I think we’ve basically lost the life we had. Just recognizing that it’s a loss, that it’s disappeared, that it’s gone. It’s like watching a death happen in slow motion. You know if something happens to someone you might mourn and then its passed. This is the longest mourning you can imagine. Because you’re just watching loss, in slow motion”.* (1021)  “*They ( formal care support) never gave him five days. But if they had given five days I would have been able to stay at work longer. So in the end I had to give ups work to, to um look after him full time*”. (1071)  “*I had a carer for 5 hours the other day ‘cause I wanted to go to [city] with my daughter. But you see that’s what? Seventy five pounds before you’ve even got out the door! So it, none of it comes, I mean I’m not moaning about the money but none of it comes cheap. And if you haven’t got many pennies you do have to consider it*”. (1059)  “*You know, we’ve both had, independently, we had lives where we’ve done a lot and then since we got together, we did a lot in the first sort of ten years or so. So, ok, the last five, six, seven years have been very restrictive but at least we’ve got the memories of things that we have done”.* (1059)  *“There are lots of things I miss. I miss being able to sleep through the night without having to wake up several times to help (persons name) up, […] It’s actually the independence to be a person. I’ve lost my profession, because I no longer work, and it I a profession in which I could have worked probably until the day I die, because I worked independently, so I’ve lost that. I’ve lost connections with people who are pertinent in that field, umm (coughs) I‘ve stopped writing, I’ve stopped, you know, so yes I ‘ve lost a lot of that*”. (1021)  “*We were still doing it (voluntary work), she was diagnosed in 2003, and we soldiered on, and I could see this is getting more and more difficult. When we got to 2007, I said to J, “look this is not working now, it’s taking you so long to get ready to go out everywhere. So I said to (persons name) “do you know what with your Parkinson’s and my you know heart thing I think we’d better stop. But then she was very disappointed, extremely disappointed, and didn’t want to stop to start with, but she could see that what I was saying made sense. So we stopped it in 2007 or 2008, 7, 2007, having done yeah hundreds of job in eleven years. I mean we haven’t had any holidays since 2007*”. (1064)  “*The holidays that we used to have were, we’d go off you know and sort of stay in a little B&B and have walking holidays. That was our main holidays, were walking holidays and things like this. Which obviously you can’t do*”. (1094)  *“Well, its change again, you see? I generally make my own decisions now and then run them past him”* [1059] |
